# Multi-Omics Joint Analysis of Molecular Mechanisms of Compound Essential Oils Inhibiting Spoilage Yeast in Paocai

**DOI:** 10.3390/foods14111998

**Published:** 2025-06-05

**Authors:** Xinyi Wu, Zhiyan Zhu, Hao Tian, Li Liu, Xuerui Li, Jun Pan, Yifan Hu, Zhirui Niu, Hanmo Wang, Xiuwei Liu

**Affiliations:** 1 Agro-Products Processing Research Institute, Yunnan Academy of Agricultural Sciences, Kunming 650223, China; ssslwxy@163.com (X.W.); lxr@yaas.org.cn (X.L.);; 2 Honghe Hopen Food Co., Ltd., Honghe 651400, China; 3 Yunnan Institute of Product Quality Supervision and Inspection, National Tropical Agricultural By-Products Quality Inspection and Testing Center, Kunming 650223, China

**Keywords:** *Pichia manshurica*, compound essential oils, transcriptomics, metabolomics

## Abstract

*Pichia manshurica* is the main spoilage fungus that causes the deterioration of paocai. Our previous study found that the compound essential oils (CEOs) of lemon, lemongrass, and nutmeg had a good inhibitory effect; however, the antimicrobial mechanism was unknown. In order to elucidate the mechanism of action of the CEO in inhibiting *P. manshurica*, transcriptomics and metabolomics were used for joint analysis. The results showed that the minimum inhibitory concentration (MIC) of *P. manshurica* was 2 µL/mL, and the combined multi omics analyses indicated that the treatment of the CEO disrupted the ABC transporters, glycophospholipid metabolism, and nucleotide metabolism of *P. manshurica*, leading to the disruption of the integrity of *P. manshurica* cell wall and cell membrane, resulting in energy and metabolic dysfunction, and ultimately achieving the effect of inhibiting *P. manshurica*. The results of this study provided new insights into the mechanism of *P. manshurica* inhibition by CEOs, and provide a reference basis for the development of food-related bacteriostatic agents by CEOs.

## 1. Introduction

The Zhou Dynasty in China is when paocai first appeared, more than 3000 years ago [1]. Paocai is a traditional fermented vegetable product that is widely made and consumed in China and around the world [2]. The primary method for making paocai is to soak vegetables in brine before allowing them to ferment at room temperature [3]. A number of elements, such as raw materials, microbiota, and processing techniques, influence the quality of fermentation, an old processing technology [4]. One of the frequent issues impacting the paocai industry and the health of consumers is the development of spoilage bacteria during fermentation [5]. Yeast is the main spoilage bacterium that causes fermented vegetable products to spoil. Studies have shown that common spoilage yeasts for kimchi include *Pichia*, *Kazakh*, *Rhodotorula*, *Candida*, *Yarrowia*, and *Debagaea* [6]. Our research group found that *Pichia manshurica* is one of the main spoilage bacteria causing the deterioration of paocai quality, which is consistent with the conclusions drawn by previous studies on fermented cucumbers [7]. Food safety issues and large financial losses can result from spoilage bacteria [8]. Therefore, in the paocai sector, inhibiting rotting microorganisms is a critical issue that requires immediate attention.

The food industry uses synthetic preservatives extensively to maintain food safety and quality by limiting the growth of bacteria that cause spoiling and disease. However, the food industry is searching for natural substitutes due to consumer desire for natural food additives and concerns about the safety of synthetic preservatives [9]. In addition to their many antibacterial properties, plant essential oils show promise as food preservatives [10]. They work well to prolong the shelf life of juices, dairy products, and baked goods, and they aid in the preservation of a variety of food items [11]. In the food and pharmaceutical industries, compounded essential oils (CEOs) made from cinnamon and clove essential oils have been suggested as safe and natural antifungal blends due to their synergistic antimicrobial properties [12]. Clove oil and lemongrass essential oils, as well as cinnamon bark and lemongrass essential oils, have also demonstrated synergistic antimicrobial effects [13]. This indicates that the combination of CEOs has a significant synergistic effect on food antimicrobials. In the previous study of our group, it was found that the CEOs of lemon, lemongrass, and nutmeg essential oils, applied in paocai, could significantly inhibit the growth of spoilage fungus *P. manshurica*. However, there are fewer studies on the inhibition of the essential oils on the spoilage bacteria of paocai, and the study on the mechanism of inhibition of the CEO has not yet been reported.

Thus, the inhibitory impact of CEOs on the paocai rotting bacterium *P. manshurica* was the main focus of this investigation. The inhibitory mechanism of CEOs was examined in conjunction with transcriptomics and metabolomics. The study’s findings can offer a solid theoretical and scientific foundation for the synthesis of essential oils that function as a bacterial inhibitor in paocai.

## 2. Materials and Methods

### 2.1. Reagents

Lemon essential oil, lemongrass essential oil, and nutmeg essential oil were purchased from Florihana, Provence, France. All culture media used in this study were obtained from Hopebiol Company, Qingdao, China.

### 2.2. Cells and Culture Conditions

Spoilage fungus was isolated from paocai and identified as *P. manshurica* through 18S rDNA sequencing, compared with known Genbank sequences, and used in this experiment. The strain was activated in Yeast Extract Peptone Dextrose Medium (YPD) for cultivation. After the cells reached the logarithmic growth phase, they were cultured and centrifuged at 4000 rpm for 5 min. The cell suspension was then adjusted to 10^6^ CFU/mL with phosphate-buffered saline (PBS) for subsequent testing.

### 2.3. Inhibitory Activity of Compounded Essential Oils Against P. manshurica

CEOs of lemon, lemongrass, and nutmeg were combined in a volume ratio of 1:1:1 to produce CEOs. The antibacterial activity of the essential oils was assessed using the twofold dilution method to obtain the minimum inhibitory concentration (MIC) and minimum bactericidal concentration (MBC). According to Cai et al. [14], Tween 20 (0.1%) solution was used to dilute the CEO to a range of concentrations from 6.4 to 0.2 µL/mL. The *P. manshurica* bacterial solution was then put in 96-well plates with a YPD medium. Following 6, 12, 18, 24, 30, 36, 42, and 48 h of incubation at 30 °C, growth was assessed by spectrophotometry at 600 nm.

### 2.4. The Morphological Changes Caused by CEOs

According to Yu et al. [15], changes in cell morphology were detected using scanning electron microscopy (SEM) (Hitachi, Tokyo, Japan). When the YPD medium was in the logarithmic growth phase, MIC-CEO was introduced. The cell suspension was harvested by centrifugation at 4000 rpm for 5 min after being incubated for 4 h at 30 °C with constant agitation at 200 rpm. It was then rinsed twice with phosphate-buffered saline (PBS, pH 7.4). After adhering to slides, the diluted microspheres were fixed for five hours at 4 °C in 2.5% (*v*/*v*) glutaraldehyde. Following that, the cells on the slides were successively dehydrated in 20, 40, 60, 80, and 100% ethanol. The cell-containing slides were then sputter-plated with gold after being freeze-dried for 12 h at −40 °C. As a control, YPD cells devoid of the CEO were employed. A Hitaci SU8010 scanning electron microscope (Hitachi SU8010, Japan) was used to view the morphological alterations of the cells.

### 2.5. RNA-Seq Analysis

Trizol Reagent (Invitrogen Life Technologies, Carlsbad, CA, USA) was used to extract *P. manshurica*’s total RNA. A NanoDrop spectrophotometer (Thermo Scientific, Waltham, MA, USA) was employed to measure the purity of the RNA. Oligo (dT) was used to separate the mRNA from the total RNA. Following the production of the cDNA, the mRNA was extracted by adding a fragmentation buffer and choosing the right conditions to randomly split the mRNA into tiny fragments of roughly 300 bp. The final library was purified by adding adapter sequences, sorting the purified fragments, and then using the sorted products for PCR amplification and purification. Using the NovaSeq X Plus platform (Illumina, San Diego, CA, USA), sequencing libraries were sequenced. Six replicates of each treatment were used in the RNA-Seq experiments [16,17].

### 2.6. LC-MS/MS Analysis

A Thermo Vanquish ultra-high performance liquid chromatograph (UHPLC) in conjunction with a Q Exactive HF-X combined quadrupole Orbitrap mass spectrometer (Q Exactive HF-X) served as the platform for the LC-MS analysis. Chromatographic conditions were as follows: mass spectrometry was used to detect the 2 µL of sample that had been separated on an Accucore C30 column (100 mm × 2.1 mm i.d., 2.6 µm; Thermo). The mobile phases were 2 mM ammonium acetate acetonitrile/isopropanol/water (10/88/2) (containing 0.02% formic acid) and 10 mM ammonium acetate 50% acetonitrile aqueous solution (containing 0.1% formic acid). Separation gradient was as follows: within 0–4 min, mobile phase B increased from 35% to 60%; within 4–12 min, it increased from 60% to 85%; within 12–15 min, it increased from 85% to 100%; within 15–17 min, it remained at 100%; during 17–18 min, it dropped from 100% to 35%; and during 18–20 min, it remained at 35%. The column temperature was 40 °C, and the flow rate was 0.40 mL/min. Conditions for mass spectrometry were as follows: sample mass spectrometry signals were acquired within the 200–2000 *m*/*z* mass scanning range using both positive and negative ion scanning modes. The sheath gas was set at 60 psi, auxiliary heating gas at 20 psi, the ion source heating temperature at 370 °C, the cyclic collision energy 20–40–60 V, the positive mode ion spray voltage at 3000 V, and the negative mode ion spray voltage −3000 V [18,19].

### 2.7. Bioinformatics Analysis

LC/MS raw data were pretreated using Progenesis QI (version 3.0) software, exporting a 3D CSV matrix containing sample details, metabolite names, and mass spectral intensities. Internal standards and known false positives (e.g., noise, column bleed, reagent peaks) were removed, followed by deduplication and peak pooling. Following the library search, the matrix data was submitted for data analysis on the Meggie BioCloud platform (https://cloud.majorbio.com, accessed on 18 April 2024) [20]. The preprocessed matrix files were subjected to difference analysis. Principal component analysis (PCA) and orthogonal least partial squares discriminant analysis (OPLS-DA) were conducted using the R software package rolls (Version 1.6.2). Additionally, multiplicative analysis of variance and the student’s *t*-test were conducted. The OPLS-DA model’s variable weight values (VIP) and the student’s *t*-test *p*-value were used to select significant metabolites, metabolites with VIP > 1 and *p* < 0.05 were classified as such.

All samples’ clean data for RNA-Seq datasets were assembled from scratch using Trinity after adapters and low-quality reads were discarded. The assembly results were then examined using the program BUSCO (Benchmarking Universal Single-Copy Orthologs, http://busco.ezlab.org, accessed on 18 April 2024) to assess how well the assembly results were optimized. The per kilobase per million mapped reads (RPKM) method was used to standardize the gene expression levels [21]. The R package DEGseq was used to calculate differentially expressed genes [22]. GO (Gene Ontology, http://geneontology.org, accessed on 18 April 2024) and KEGG (Kyoto Encyclopedia of Genes and Genomes, http://www.kegg.jp, accessed on 18 April 2024) were used to annotate all genes that were differentially expressed.

## 3. Results

### 3.1. Effect of CEOs on P. manshurica MICs

**Figure 1:**
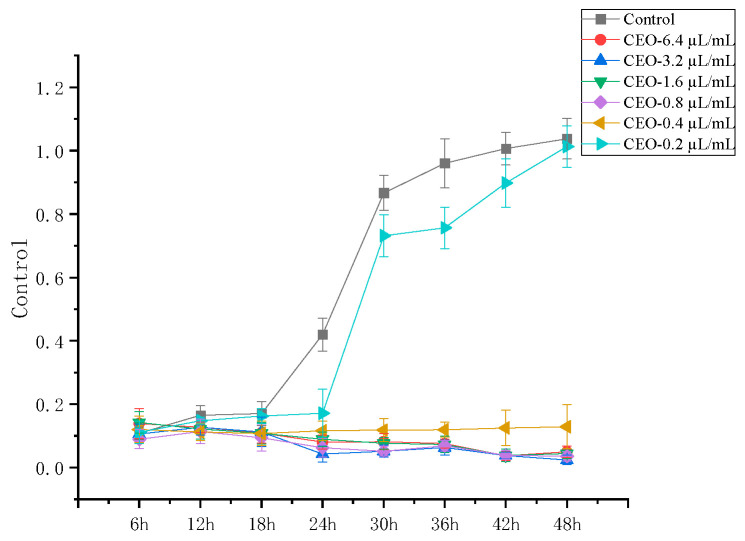
Antifungal activity of CEOs against *P. manshurica*.

The quality of paocai may suffer as the yeast population grows, particularly in the later phases of fermenting [23]. In line with the results of earlier research on fermented cucumber, *P. manshurica*, one of the yeasts that causes paocai to deteriorate, was isolated from paocai in this study [7]. The antifungal activity of the CEO was quantitatively assessed using the MIC values. At every stage of the trial, *P. manshurica*’s OD values remained relatively constant following exposure to the CEO, suggesting that the CEO possessed fungicidal properties. *P. manshurica* had a MIC of 0.4 µL/mL and an MBC of 0.8 µL/mL (Figure 1).

### 3.2. Effect of CEOs on P. manshurica Cell Morphology

Because of their biological potential and relative safety for food and agricultural products, plant essential oils provide good substitutes for synthetic fungicides. With their high bioactivity, lemon, nutmeg, and lemongrass essential oils show promise for food preservation [24,25,26]. Nevertheless, there have been few reports on the antifungal activity and mechanism of the three essential oils when combined with yeast.

**Figure 2:**
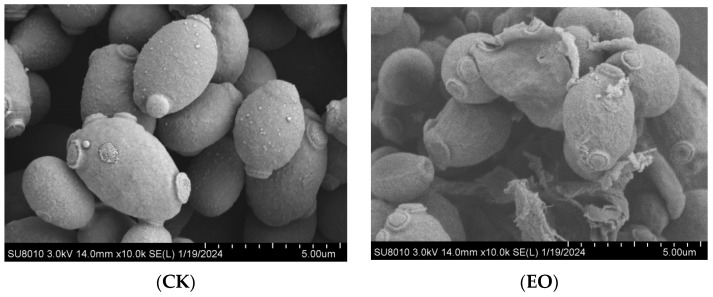
The cell morphology treatment with the MIC CEO of *P. manshurica* by SEM (CK: control group; EO: CEO-treated group).

Cells typically undergo morphological alterations following antimicrobial medication therapy. According to the results of scanning electron microscopy, *P. manshurica* treated with the CEO had a considerably different cell morphology than samples that were not treated. The CK group’s cells morphology, which included distinct cell margins, remained normal. On the other hand, the CEO-treated *P. manshurica* cells surface displayed rupture and deformation, some cells showed crumpled and collapsed, and the contents of the cells overflowed with hazy cell borders (Figure 2).

### 3.3. Transcriptome Sequencing Analysis

#### 3.3.1. Data Processing and Analysis

The Illumina sequencing platform collected 520,333,406 raw reads, and after data filtering, sequencing error rate checking, and guanine (G) and cytosine (C) content distribution checking, 517,784,948 (99.51%) of them were clean reads. All the samples had Q30 quality scores of 95% or higher, and the GC content was 45% or higher. These findings suggest that the sequencing quality is adequate for further examination.

#### 3.3.2. Diversity Analysis

**Figure 3:**
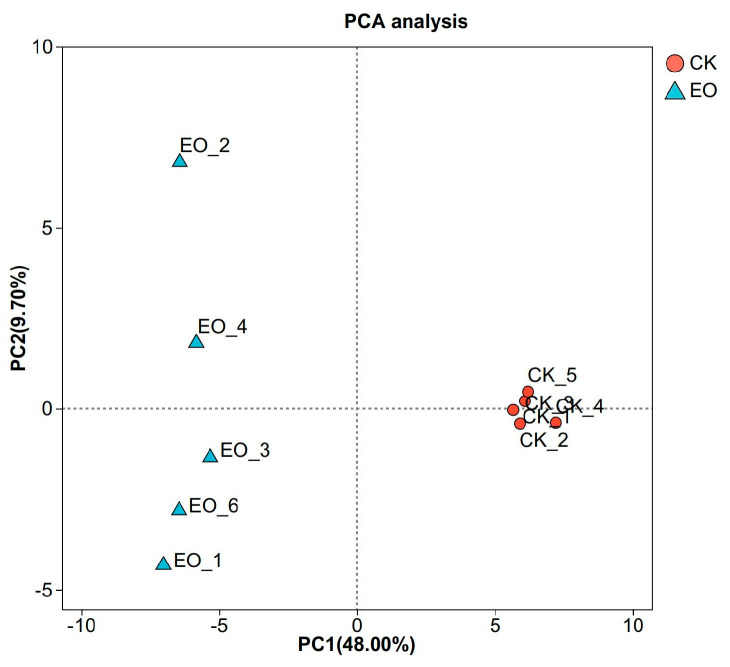
Principal component analysis (PCA) based on FPKM. (CK: control group; EO: CEO-treated group).

The correlation of gene expression levels between samples is an important index to test the reliability of the experiment. As shown in Figure 3, six biological replicate samples from different treatments were clustered together based on the analysis of Fragments Per Kilobase of exon model per Million mapped fragments (FPKM) values, indicating that the data from this sequencing can be used for subsequent biological analyses, and the samples from the treatment and control groups are separated, which is an indication of the difference in gene expression levels between the two groups, indicating that the compound essential oil treatment has a significant effect on *P. manshurica* gene expression.

#### 3.3.3. DEGs

**Figure 4:**
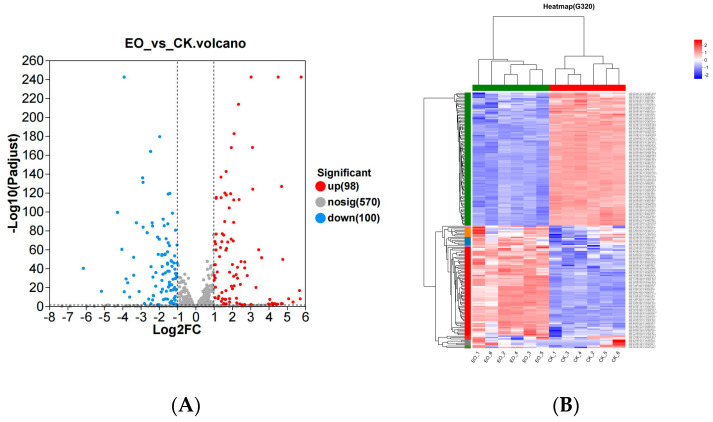
DEGs analysis. (**A**) Volcanic maps of all DEGs. (**B**) Clustering heat map of DEGs. Columns represent samples, and rows represent genes.

The DESeq2 program was used to examine the differences in gene expression, and the criteria for screening DEGs were |log2 (fold change)| > 0 and *Padj* < 0.05. The distribution of differential genes between the treatment and control groups is displayed in the volcano plot (Figure 4A), where down-regulated genes are shown in green and up-regulated genes in red. In both the treatment and control groups, a total of 198 gene fragments were found; in the treatment group, 100 of these were down-regulated, while 98 were up-regulated.

Hierarchical clustering was subsequently employed to group the DEGs, enabling the clustering of genes or samples displaying similar expression patterns, as depicted in the heat map (Figure 4B). The analysis revealed that the samples exhibited consistent responses after adding the compound essential oil blend. Nevertheless, further investigation is warranted to elucidate the specific impact of cinnamon essential oil (CEO) on the activity of these DEGs.

#### 3.3.4. GO Enrichment Analysis

**Figure 5:**
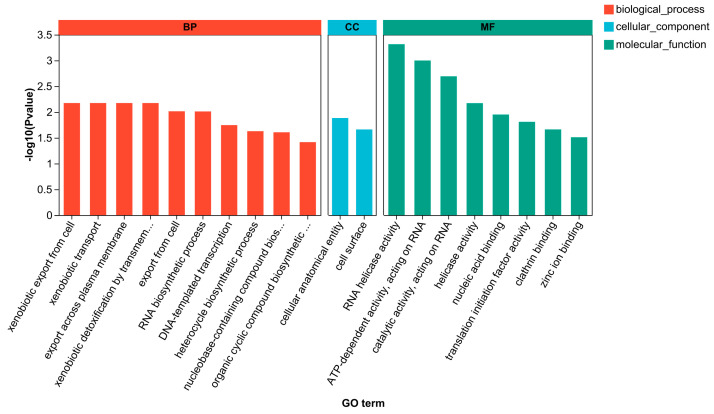
GO enrichment analysis of DEGs.

A widely recognized gene function classification system, the GO database offers a collection of regularly updated standard vocabularies to thoroughly explain the functional characteristics of genes and gene products in living things. Three main categories—Molecular Function (MF), Cellular Component (CC), and Biological Process (BF)—are present in the database. These categories described the molecular functions that a gene product may perform, as well as the cellular environment and biological processes in which it is involved. Following CEO treatment, four hundred and eighty-one DEGs were enriched to twenty-four terms, including ten BP, two CC, and twelve MF. The TOP 20 enrichment results are displayed in Figure 5. Cellular anatomical entities (GO:0110165, 120), cell surfaces (GO:0009986, 4), organic cyclic compound biosynthesis (GO:1901362, 29), heterocycle biosynthesis (GO:0018130, 28), nucleic acid binding (GO:0003676, 56), and ATP hydrolysis activity (GO:0016887, 29) were the categories for which the top two DEGs were found.

#### 3.3.5. KEGG Enrichment Analysis

**Figure 6:**
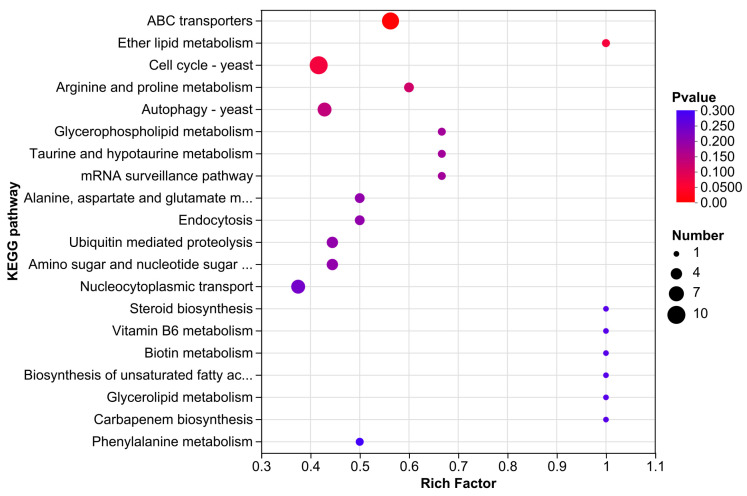
KEGG enrichment analysis of DEGs.

The KEGG database incorporates information from genomes, chemical compounds, biochemical systems, and other sources to provide a systematic examination of the metabolic pathways of gene products in cells and their roles. Following CEO treatment, a total of 180 DEGs were enriched to 72 pathways; Figure 6 displays the TOP 20 enrichment results. Cell cycle (map04111), ABC transporters (map02010), meiosis (map04113), MAPK signaling pathway (map04011), cofactor biosynthesis (map01240), pyruvate metabolism (map00620), aminoacyl-tRNA biosynthesis (map00970), nucleocytoplasmic transport (map03013), oxidative phosphorylation (map00190), and autophagy (map04138) were the pathways that contained the top 10 DEGs.

### 3.4. Metabolome Analysis

**Figure 7:**
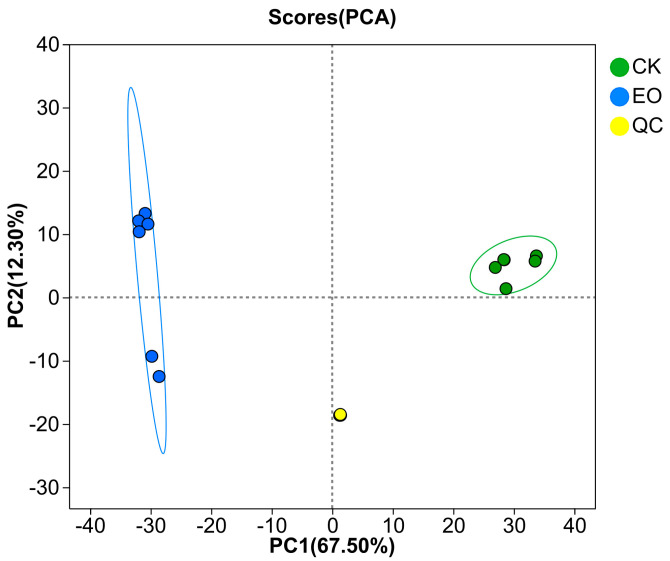
PCA analysis of all samples (green dots: CK, control group; blue dots: EO, CEO-treated group; yellow dots: QC, quality control).

This study used LC-MS to examine the impact of CEOs on *P. manshurica* metabolism. PCA analysis of twelve data points from six biological replicates revealed clustering of replicates and strong separation between the two groups, as Figure 7 illustrates.

**Figure 8:**
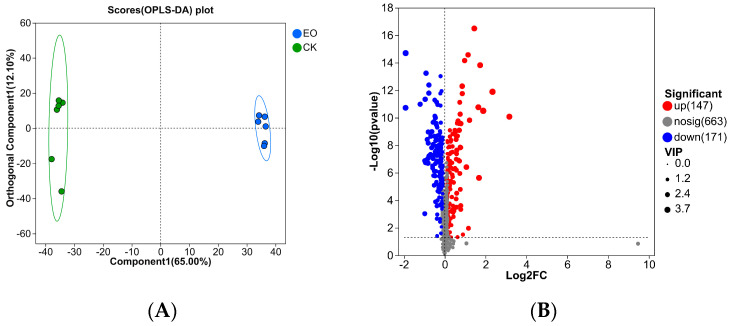
Data analysis of metabonomics. (**A**): OPLS-DA analysis. (**B**): Volcano map of all metabolites detected.

The metabolic profiles of all samples were compared using OPLS-DA to evaluate the changes in metabolic profiles created by the compounded essential oils in order to ascertain the differences in *P. manshurica* metabolites between the CEO treatment and control groups (Figure 8A). For the OPLS-DA model, the R^2^X (cum) and R^2^Y (cum) values represent the cumulative explained variance rates. The closer these values are to 1, the more satisfactory the model’s interpretability and predictability become [26]. Consequently, the model has a good predictive value (R^2^X cum = 0.997) and a strong fitness (R^2^Ycum = 0.999). The two sample groups were distinguished on the score plot, suggesting that the compounded essential oils’ antibacterial action was noticeable at the metabolic level.

Significant differences between the two groups were determined using the student’s *t*-test, and metabolites with a VIP value > 1.0 and a *p* < 0.05 were deemed differential metabolites (Figure 8B). In comparison to the control group, 147 of the 318 differential metabolites that were tested showed up-regulation and 171 showed down-regulation in the compound essential oil treatment group.

**Figure 9:**
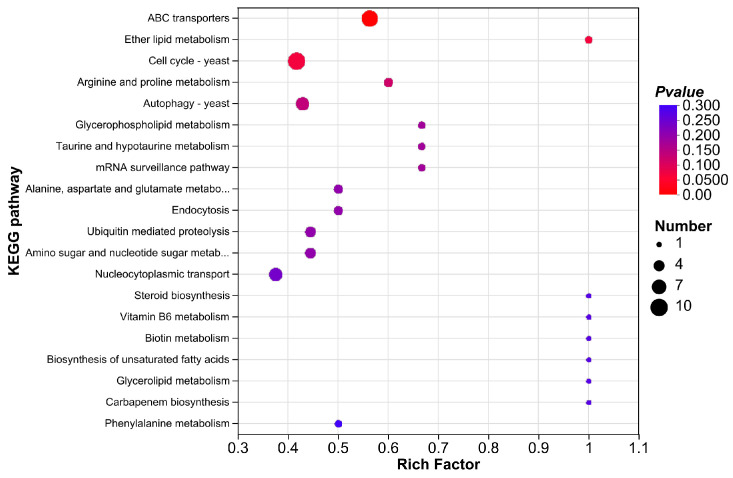
KEGG enrichment analysis of differential metabolites.

Pathway analysis was performed to examine the interference of *P. manshurica*’s metabolic network following CEO treatment, based on the differential metabolites previously described. Of the seventy-one metabolic pathways that were found, six were highly enriched (*p* < 0.05). These pathways included the metabolism of purines, nucleotides, ABC transporters, glycerophospholipids, biosynthesis of cofactors, and arachidonic acid (Figure 9).

### 3.5. Multi-Omics Association Analysis

**Figure 10:**
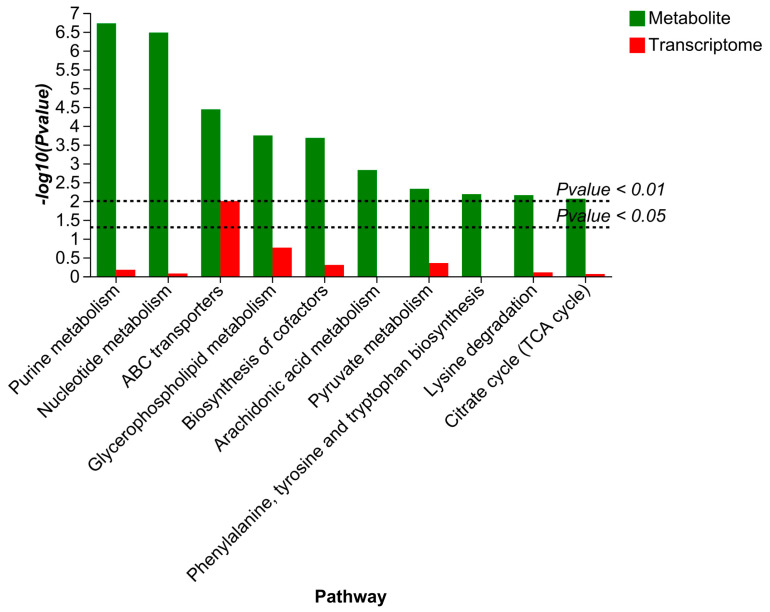
KEGG enrichment analysis.

Based on the findings of the enrichment analysis of differential genes and metabolites, histograms were plotted. The metabolic pathways were represented by the horizontal coordinates in Figure 10, the DEG enrichment *p*-values by the red columns, and the differential metabolite enrichment *p*-values by the green columns. The ABC transporters pathway was the only pathway with significant enrichment of metabolites (*p* < 0.05) after treatment with the CEO, in both the control and treated groups. This suggested that the ABC transporters pathway may be the primary pathway for the CEO’s inhibition of *P. manshurica*. Other pathways with significant enrichment of metabolites (*p* < 0.05), but not of differential genes (*p* > 0.05), include glycerophospholipid metabolism, pyruvate metabolism, biosynthesis of cofactors, purine metabolism, lysine degradation, nucleotide metabolism, and citrate cycle (TCA cycle). This may also be the primary pathway for the CEO’s inhibition of *P. manshurica*. The complexity of the gene–function relationship makes it more challenging to explain transcriptomics [27]. In the meantime, metabolites—substances that organisms generate or consume—were found near the conclusion of the control of life activities and are closest to the phenotype, serving as a crucial link between genes and phenotypes. Metabolites will become more visible and subtle gene alterations will be magnified many times at the metabolic level [28].

## 4. Discussion

Though the use of the three essential oils in a compounded form has not been documented, lemon essential oil, lemongrass essential oil, and nutmeg essential oil have good antimicrobial effects against a variety of foodborne pathogens, including *Salmonella typhimurium*, *Escherichia coli*, *Staphylococcus aureus*, *Klebsiella pneumoniae*, and *Enterococcus faecalis* [29,30]. Our results indicate that the MIC and MBC of the CEO against *P. manshurica*, were 0.4 µL/mL and 0.8 µL/mL, respectively. To further analyze the antibacterial activity of the CEO against *P. manshurica*, we plotted the growth curves of *P. manshurica* treated with different concentrations of the CEO. These curves further confirmed that the CEO exhibited a concentration-dependent antibacterial effect against *P. manshurica*. In addition, SEM results also showed that the treatment of CEO resulted in deformation and rupture of *P. manshurica* cell surfaces, which ultimately led to cell death, proving that the CEO had a good bacteriostatic effect.

The collaborative analysis’s findings imply that ABC transporters might be the primary mechanism by which the CEO inhibits *P. manshurica*. By regulating phospholipid asymmetry, ABC transporter proteins can affect plasma membrane function and are significant determinants of membrane function. By disrupting cellular membranes, Kalli et al. demonstrated that glabridin and wighteone prevented food deterioration in the yeast *Zygosaccharomyces parabailii* [31]. Thus, based on our observations and experimental data, it is postulated that treatment with CEOs alters the ABC transporter pattern in *P. manshurica*. This disruption likely destabilizes the intracellular and extracellular balance, placing stress on the cell membrane. As a result, the membrane begins to rupture, compromising cellular integrity and leading to the leakage of vital components and influx of harmful substances. Ultimately, these effects culminate in the organism’s demise, as the cells can no longer sustain their essential functions. This aligns with prior research showing that essential oils and their components can damage the cell membranes of foodborne yeasts, causing cell death [32].

Furthermore, the collaborative analysis’s findings identified a number of additional, more plausible courses of action [33]. Zheng et al. showed that propolis caused disruption of the metabolism of glycerophospholipids in *Clostridium perfringens*, which resulted in bacterial inhibition [34]; Zhao et al. showed that Longistylin A inhibits *Neisseria* spp. by interfering with the metabolism of glycerophospholipids, which are important components of lipoproteins, and disrupting their metabolism changes the function of lipoproteins [35]. Building upon these findings, it is plausible to hypothesize that one of the mechanisms by which CEO inhibits *P. manshurica* could involve the disruption of glycerophospholipid metabolism. Such interference could lead to structural and functional alterations in the cell membrane, compromising the integrity of the cell and ultimately resulting in its demise.

A component of cell wall polysaccharides called peptididoglycan is essential for preserving bacterial shape and fending off osmotic stress. As a crucial part of peptidoglycans, nucleotides aid in the regular growth and division of bacterial strains [36]. Furthermore, lysine is one of the first cellular constituents to experience external stress and is also a crucial part of peptidoglycan [37]. Thus, it is hypothesized that treatment with CEOs may disrupt the metabolism of lysine and nucleotides in *P. manshurica*. These disruptions could compromise the cell wall and membrane integrity by affecting peptidoglycan synthesis and stability. As a result, the cell wall becomes less robust, and the membrane more permeable, leading to cellular leakage and susceptibility to external harm. These changes collectively explain the antibacterial effects of CEO against *P. manshurica*, as the compromised cell structure inhibits the bacterium’s survival and growth.

The strain enters the TCA cycle after using the glycolytic pathway to convert glucose to pyruvate. An essential metabolic route for all aerobic organisms, the TCA cycle serves as the primary core conduit connecting the majority of separate metabolic pathways [38]. By inhibiting the TCA cycle, Ning et al. discovered that sucrose laurate had an antibacterial impact on *Bacillus cereus* [39]. By altering pyruvate metabolism and the TCA cycle, Liang et al. discovered that the combination antibacterial activity of thymol and cinnamonaldehyde against *Listeria monocytogenes* on steamed chicken breast meat was accomplished [40]. Drawing on these findings, it is plausible to hypothesize that the demise of *P. manshurica* following treatment with CEOs could be attributed to the disruption of the TCA cycle. This disruption likely stems from the disturbance of pyruvate metabolism, a critical step preceding the TCA cycle. By interfering with this metabolic pathway, CEOs may effectively starve the bacterium of the energy and biosynthetic precursors necessary for its survival and growth, ultimately leading to its inhibition or death. This result indicated that *P. manshurica* perished due to the disruption of the TCA cycle, which resulted from disturbances in pyruvate metabolism during CEO therapy.

## 5. Conclusions

Using a combination of metabolomics and transcriptomics research, the effects of the CEOs of lemon, lemongrass, and nutmeg on the inhibitory action of *P. manshurica* were thoroughly examined. According to the combined analysis of multiple omics, ABC transporters are the pathways that are significantly enriched in both transcripts and differential metabolites. Different metabolites, including the TCA cycle, glycerophospholipid metabolism, pyruvate metabolism, purine metabolism, lysine breakdown, nucleotide metabolism, and cofactor synthesis, are also significantly concentrated. By changing the makeup and functionality of cell walls and membranes, CEOs can effectively prevent *P. manshurica* from growing, which results in energy and metabolic inefficiencies, according to the study’s findings. These results provide new insights and lines of inquiry into the molecular-level inhibitory potential of CEOs.

## Data Availability

The original contributions presented in this study are included in the article. Further inquiries can be directed to the corresponding author.
